# Internet use and depression among Chinese older adults: The mediating effect of interpersonal relationship

**DOI:** 10.3389/fpubh.2023.1102773

**Published:** 2023-03-03

**Authors:** Yan Nan, Yating Xie, Yuqun Hu

**Affiliations:** ^1^Department of Social Security, School of Public Policy and Administration, Xi'an Jiaotong University, Xi'an, China; ^2^Research Center for Social Governance Innovation, Henan Agricultural University, Zhengzhou, China

**Keywords:** Internet use, depression, older adults, interpersonal relationship, Chinese

## Abstract

The number of elderly Internet users has increased significantly in the past few years, and the Internet has greatly changed the way that older adults access information and communicate. Generally, those who regularly use the Internet may expand their range of interpersonal interactions, which has positive effects on their health. Depression is the leading cause of ill health, and is closely related to people's mental health. We sought to investigate whether internet use help reduce depression level among older adults. Using data from the 2020 China Family Panel Studies (CFPS), a total of 4,714 respondents were included to explore the effects of Internet use on the elderly's depression levels in China, along with the mediating role of interpersonal relationship in the above relationship. Regression results indicated that Internet use significantly reduced depression levels among the elderly. Further analysis showed that different Internet usage had different effects on depression among the elderly. Wechat chatting, video browsing, and online shopping were positively correlated with lower levels of depression. However, playing online games and online learning did not predict reduced levels of depression. Moreover, interpersonal relationship mediated the relationship between Internet use and depression levels. Internet use was associated with a higher level of interpersonal relationship, which in turn reduced depression levels in older adults. Regarding gender and regional differences, the coefficient of Internet use for urban older adults was significantly negative at 0.001 level, while it was not significant for rural older adults. A mediating effect of interpersonal relationship between Internet use and depression levels was only found for male elderly. To reduce the level of depression and promote mental health in the course of aging, Internet use and the improvement of interpersonal relationships merit special attention.

## 1. Introduction

As China's population aging continues to deepen, an increasing number of Chinese older adults face health risks (such as loneliness, anxiety, and depression) (1). Depression, one of the most prevalent mental illnesses among the elderly, has a detrimental effect on their health (2). Data from the 2005 Chinese Population 1% Sample Survey showed that 13.6% of urban elderly population suffer from moderate or severe depression. Among the rural elderly, the proportion was 25.5% (3). The prevalence of depression increases with age, which not only impairs the mental and physical functioning of the elderly, leads to impaired cognitive function, increases the risk of diseases such as heart disease and stroke (4), but also increases the risk of suicide and death (5, 6). Therefore, exploring the mechanism of reducing depression is of great significance to improving the mental health of the elderly and promoting healthy aging.

With the rapid development of Internet technology, the number of Internet users in China was 1.032 billion as of 2021, and the Internet penetration rate reached 73.0%.While the elderly population constitutes a relatively small proportion of this group of netizens, their proportion has continued to grow. The number of elderly netizens aged 60 and above in China has reached 119 million, accounting for 11.5% of the total netizens. The Internet penetration rate of the elderly population aged 60 and above is 43.2% (7). Risk factors for poor mental health among older adults may be potentially impacted by Internet use (8). Existing research shows that Internet use is strongly associated with health and can help reduce social isolation and loneliness among older people (9, 10). But there is uncertainty about the impact of Internet use on depression among older adults. Using a systematic review of quantitative and qualitative evidence, Forsman and Nordmyr (11) reported that Internet use was not associated with depression in 40% of cases. Jun and Hey investigated the effects of Internet use on satisfaction with social relationships and depression among Korean older adults. Their research showed that Internet use did not have direct effects on depression (12). A study on the mental health of older people in England also confirmed this conclusion. Lam et al. concluded that infrequent internet use was predictive of deteriorating life satisfaction but not depression (13). However, some evidence suggested that internet use was found to decrease depression significantly (14). In a survey of retired older adults in the United States, Cotten et al. found that prior internet use reduced the probability of depression by a third in a sample of 3,000 retired adults older than 50 (15). Based on data from the 2018 China Family Panel Studies, Yang et al. found that older Chinese who used the internet reported lower depression scores (16). At the same time, the relationship between Internet use and depression may be influenced by different categories of internet usage. Using data from the 2013 and 2016 waves of the Japan Gerontological Evaluation Study, Nakagomi et al. found that using the Internet to communicate had a protective effect on the probability of developing clinical depression (17). This conclusion was reinforced by other studies. Some literature showed that using the Internet for communication with friends and family was associated with small but reliable decreases in depression. In contrast, health-related internet use was associated with small but reliable increases in depression (18). And by using the Internet to learn, work, and conduct commercial activities, the relief of depression symptoms dissipated (16).

Several studies have explored the mechanisms linking Internet use and depression among older adults. Major and potentially modifiable risk factors for depression among older adults include social isolation, reduced social contact, and lack of emotional support (19). Internet use could reduce depression by promoting older people's social engagement and expanding their social networks, thereby reducing their risk of social isolation (14, 20). However, few studies have examined the role of interpersonal relationships in the association between Internet use and depression. Interpersonal relationship refers to an individual's relationship with others, which reflects the individual's social confidence and preference (21). Interpersonal relationships can be beneficial to health by promoting processes of exploration, personal growth, and goal-striving, all of which are essential to health and wellbeing (22). If people cannot forge a desirable relationship, they will feel isolated and lonely, which will affect their mental health (23). In fact, using the Internet to communicate is one of the key channels for older people to maintain their relationships. It allows them to stay in touch with their social networks, exchange social support and improve their health (15). Additionally, older people who use the Internet will increase their social connections, be more satisfied with the connections, and have better interpersonal relationships (24, 25).

Although some researchers have explored the link between Internet use and depression among older adults and reported mixed results, evidence examining the mechanisms linking Internet use to depression is limited. This study examines how internet use affects depression among Chinese older adults and investigates whether Internet use plays a role in depression by influencing interpersonal relationships. Moreover, the digital gap between urban and rural areas or between women and men in China is huge. Therefore, this paper also considers the heterogeneity of the elderly population in China and analyze the impact of Internet use on depression among different population groups.

## 2. Data and methodology

### 2.1. Data

Our data comes from 2020 China Family Panel Studies (CFPS) conducted by the China Social Science Survey Center of Peking University. It reflects the changes in China's society, economy, population, education and health. The project is a national, large-scale, multidisciplinary social tracking exercise. The CFPS sample covers 25 provinces/municipalities/autonomous regions, with a target sample size of 16,000 households, and the respondents include all family members in the sample households. It is a reliable data source for academic research and public policy analysis. The research object of this paper was the elderly. According to the World Health Organization, elderly people are defined as those 60 years of age or older (26). So we excluded the sample of respondents under 60 years old. In addition, we removed observations with missing data on respondents' demographic information. Our final dataset consisted of 4,714 observations.

### 2.2. Variables and measures

#### 2.2.1. Dependent variable

According to the Chinese version of CES-D, participants were asked about their frequency of depressive symptoms in the past week. The CES-D scale is a commonly used measure of depression symptoms among older adults, which consists of 8 items (27, 28). Sample items include: “I feel depressed in the past week,” “I feel it takes a lot of effort to do anything in the past week,” “ My sleep is not good in the past week,” “I feel happy in the past week,” “I feel lonely in the past week,” “I live happily in the past week,” “I feel sad in the past week ,” and question “I feel that life can't go on in the past week.” Each item is scored on a scale from 1 (never) to 4 (almost every day). After the order was reversed for items with positive measures, the average score was calculated, the higher the score, the more serious the depression. Cronbach's α for the present sample was 0.78.

#### 2.2.2. Independent variable

In our study, interpersonal relationship refers to the specific state of connection between an individual and others, usually measured by the quality of the relationship. The independent variable was internet use, obtained from the question, “Do you use mobile devices or computers to surf the internet?”. The available answers were “Yes” and “No.” we assigned 1 to “Yes” and 0 to “No.”

#### 2.2.3. Control variables

Referring to the relevant literature (16, 29, 30), we controlled the following variables: gender (Gender), marital status (Marriage), education attainment (Educ), household registration (Hr), retirement (Retirement), Membership of Communist Party of China (Party), self-assessed socio-economic status (Status), self-assessed social class (Class) and physical health (Health). All control variables and their definitions are shown in Table 1.

**Table 1:** Basic characteristics for variables.

**Variables**	**Category**	***N***	**Percentage or mean (SD)**
**Dependent variable**			
Depression scores from CES-D8		4,714	1.69
**Independent variable**			
Internet use	Yes	1,082	22.95%
	No	3,632	77.05%
**Mediating variable**			
Interpersonal relationship	4,714	7.39
**Control variables**			
Gender	Female	2,240	47.52%
	Male	2,474	52.48%
Age	4,714	68.12
Marital status	Married or living with a spouse	3,949	83.77%
	Divorced or widowed	765	16.23%
Education attainment	No formal education	1,860	39.46%
	Primary school	1,059	22.46%
	Junior high	1,033	21.91%
	Senior high	625	13.26%
	College or higher	137	2.91%
Household registration	Rural	2,345	49.75%
	Urban	2,369	50.25%
Retirement	Retired	2,545	53.99%
	Unretired	2,169	46.01%
Member of communist party of China	Yes	644	13.66%
	No	4,070	86.34%
Self-assessed socio-economic status	4,714	3.19
Self-assessed social class	4,714	3.48
Physical health	4,714	2.16

#### 2.2.4. Mediating variable

The mediating variable was interpersonal relationship. According to Liu's research (21), interpersonal relationship refers to the specific state of connection between an individual and others, usually measured by the quality of the relationship. In this study, interpersonal relationship refers to the universal contact between individuals and others, including both the online relationship and the relationship in real life. Because the use of the Internet may not only have an impact on the online interpersonal relationship, but also may have an impact on the real interpersonal relationship. We measured the mediating variable with the question, “How about your relationship with others?”. The available scores ranged from 0 (“Very poor”) to 10 (“Very good”).

### 2.3. Data analysis

In this study, all analyses were conducted in Stata 14.0 software. Firstly, we established ordinary least squares (OLS) model as a benchmark model to test the effect of Internet use on depression among the elderly. Secondly, we established propensity score matching (PSM) method to overcome the selective bias of Internet use, and further verified the conclusions of the OLS model to see whether this effect continues to exist. Thirdly, we constructed a mediation model with a bootstrap sample of 5,000 to examine the role of interpersonal relationships in the association between Internet use and depression. Finally, we examined the heterogeneous effect of Internet use on depression in different samples.

## 3. Empirical results

### 3.1. Sample details

As shown in Table 1, the mean depression score among the 4,714 older adults was relatively low (mean = 1.69). Of all respondents, 2,240 (47.52%) were women, and 2,474 (52.48 %) were men. The depression score of female seniors was 1.78, slightly higher than that of male seniors (1.62). The average age was 68.12. About 83.77% were married or living with their spouses. The majority of them were low educated, with 16.17% having a high school degree or higher. Nearly half (49.75%) of the respondents came from rural areas. The proportion of retired elderly people was 53.99%. Among the respondents, there were 644 Chinese Communist Party members. The average scores of self-assessed socio-economic status and self-assessed social class were 3.19 and 3.48, respectively.

### 3.2. Benchmark regression results

Model 1 in Table 2 showed the null model, reporting the results without control variables. And model 2 reported the results with control variables. As Model 1 showed, the coefficient of Internet use was significant and negative at 0.001 level. The result indicated that Internet use could reduce depression levels among the elderly in China. This conclusion was still supported when control variables were included.

**Table 2:** The benchmark regression results on internet use and depression.

**Variables**	**Model 1**	**Model 2**
Internet use	−0.171^***^(0.019)	−0.070^***^(0.020)
Gender		−0.087^***^(0.016)
Age		−0.004^*^(0.001)
Marital status		−0.155^***^(0.021)
Education attainment		−0.037^***^(0.008)
Rural/urban residence		−0.124^***^(0.016)
Retirement		−0.060^***^(0.017)
Party member status		−0.042(0.023)
Self-assessed socio-economic status		−0.045^***^(0.008)
Self-assessed social class		−0.033^***^(0.008)
Physical health		−0.167^***^(0.008)
Constant	1.734^***^(0.009)	2.898^***^(0.108)
Observations	4,714	4,714
R-squared	0.016	0.178

^*^p < 0.05, ^***^p < 0.001; Standard errors are in parentheses.

As Model 2 in Table 2 showed, gender, age, marital cohabitation, education attainment, household registration, retirement, self-assessed socio-economic status, self-assessed social class, and physical health had varying degrees of influence on the level of depression among the elderly. Overall, the regression coefficients of gender, marital cohabitation, education attainment, household registration, retirement, self-assessed socio-economic status, self-assessed social class, and physical health were significant at 0.001 level. The effect of age on depression was significant at 0.05 level. However, party membership status had no significant effect on depression. The results demonstrated that respondents who are male (β = −0.087, *p* < 0.001), urban residents (β = −0.124, *p* < 0.001), not retired (β = −0.060, *p* < 0.001), married or living with a spouse (β = −0.155, *p* < 0.001), were more likely to report lower depression scores. And the higher the education, the lower the level of depression. In addition, respondents with higher self-rated socioeconomic status or social class had lower depression scores. At the same time, a negative relationship existed between physical health and depression scores among older adults.

### 3.3. Endogenous treatment and robustness test

According to relevant literature (31, 32), we used PSM method to deal with the endogeneity and test robustness in model estimation. The propensity score is the conditional probability of being affected by some explanatory variable when controlling for many observable confounding variables. The PSM method can put these confounding variables into the Logit model to predict the propensity score, and then control the propensity score to alleviate the biased causal inference caused by the confounding variables and selection bias (33). In this study, we used the PSM method to overcome the selective bias of Internet use among the elderly and further verify the conclusions of the OLS model to investigate whether the relationship between variables persisted. The first step was to predict the tendency value and build a regression model with a binary dummy variable as the dependent variable. In the binary dummy variable, 1 represented the treatment group (older people using the Internet) and 0 represented the control group (older people who were not using the Internet). Independent variables were the control variables of this paper. The second step was matching based on propensity value. PSM method includes k nearest neighbor matching, absolute distance limiting propensity score, nearest neighbor matching in caliper and kernel matching, etc. The difference between matching methods is that the distance calculation method is different, and each has its advantages and disadvantages. Usually, we can choose any matching method and choose the other two matching methods to test the robustness of the results. Kernel matching, radius matching, and local linear regression matching were used in our study. The third step was to estimate the causality coefficient. The estimation results of the above methods are shown in Table 3, where ATT (Average Treatment Effect on the treated) represents the average treatment effect on the treated (the average effect of actual Internet use on depression levels among older adults). The ATT values of kernel-based matching and radius-based matching were −0.059 and −0.044, respectively (significant at the 5% level). The ATT value of local linear-based regression matching was 0.044 (significant at the 1% level). These results indicated that after controlling for endogeneity, Internet use had a negative effect on depression level. The results showed that the net treatment effects were all significantly positive, further verifying that internet use could reduce depression among older adults. The PSM test proved that the previous benchmark regression results had a certain degree of robustness.

**Table 3:** Endogenous test and robustness test results.

**Matching method**	**Kernel-based matching**	**Radius-based matching**	**Local linear regression-based matching**
ATT	−0.059^*^(0.020)	−0.044^*^(0.022)	−0.062^**^(0.021)

^*^p < 0.05, ^**^p < 0.01. Standard errors are in parentheses.

### 3.4. Mediating effect of interpersonal relationship

We further used the Bootstrap method with 5,000 bootstrap samples, proposed by Hayes (34), to test the mediating effect of interpersonal relationship, and obtain the bias-corrected 95% confidence intervals for the indirect effect and total effect.

**Figure 1:**
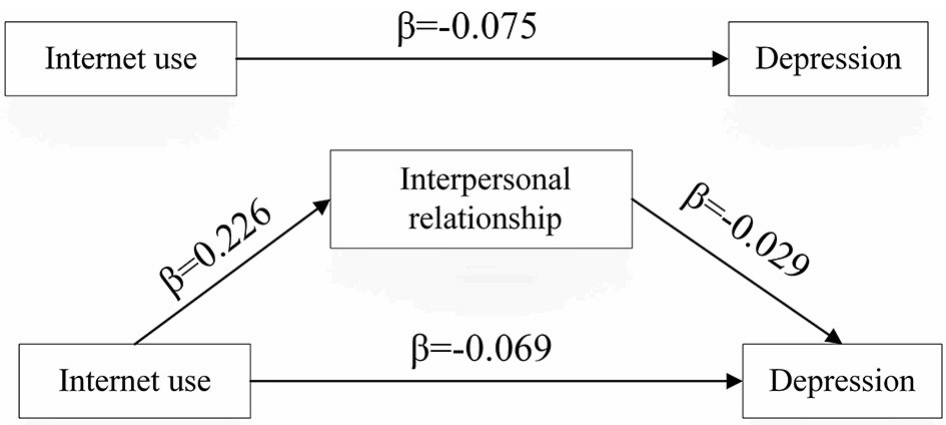
Mediating effects of interpersonal relationship in the association between Internet use and depression.

Figure 1 and Table 4 showed the mediating role of interpersonal relationship between internet use and depression level after controlling for socio-demographic characteristics. It could be seen that the coefficient of Internet use on depression was reduced after the inclusion of interpersonal relationship. The total effect of internet use on depression was −0.075 (*p* < 0.001). When perceived interpersonal relationship was included as a mediator, the effect decreased (β = −0.069, *p* < 0.001). We could see that the indirect effect of interpersonal relationship was significant at 0.01 level, and the proportion of indirect effect was 8.6%. These results indicated that interpersonal relationship partly mediated the relationship between internet use and depression among Chinese older adults.

**Table 4:** Results of mediating effect.

**Types**	**Observed coefficient**	**Bootstrap standard error**	***P*-value**	**95% Conf. interval**
Indirect effect	−0.006^**^	0.002	0.004	−0.011,−0.002
Direct effect	−0.069^***^	0.019	0.000	−0.106,−0.031
Total effect	−0.075^***^	0.019	0.000	−0.113,−0.037
The proportion of indirect effect	0.086			

Adjusting for gender, age, marital status, education attainment, household registration, retirement, party member status, self-assessed socio-economic status, self-assessed social class and physical health. ^**^p < 0.01, ^***^p < 0.001. Confidence intervals do not cross zero means the relationship is significant.

### 3.5. Heterogeneity test

This section investigated the impacts of different Internet usage on depression among the elderly. We also examined heterogeneous effects of Internet use on depression among the elderly based on regional and gender differences. The results demonstrated that using Wechat, browsing short videos, and online shopping had significant negative effects on depression. However, playing online games and online learning had no significant effect on reducing depression (Table 5). Columns (3, 4) in Table 6 showed the effect of Internet use on depression among urban and rural elderly, respectively. The coefficient of Internet use for urban older adults was significantly negative at the 0.001 level, while it was not significant for rural older adults. Columns (5, 6) in Table 6 showed the effect of Internet use on depression among female and male older adults, respectively. The results demonstrated that Internet use could significantly reduce depression among female and male older adults, but the impact was greater for females. In addition, we examined the mediating effect of interpersonal relationship between Internet use and depression among female and male older adults, respectively. The results, shown in Table 7, indicated that interpersonal relationship significantly mediates the relationship between Internet use and depression, but only among male elderly, not female.

**Table 5:** The regression results on different internet usage and depression.

**Variables**	**Model 3**	**Model 4**	**Model 5**	**Model 6**	**Model 7**
Internet use					
WeChat use	−0.082^***^(0.021)				
Short video browsing		−0.063^**^ (0.023)			
Online shopping			−0.070^*^ (0.033)		
Online games				−0.012 (0.051)	
Online learning					−0.074 (0.044)
Constant	2.386^***^ (0.105)	2.358^***^ (0.105)	2.330^***^ (0.104)	2.306^***^(0.103)	2.317^***^ (0.104)
Control variables	Yes	Yes	Yes	Yes	Yes
Observations	4,714	4,714	4,714	4,714	4,714
R-squared	0.179	0.177	0.177	0.176	0.176

^*^p < 0.05, ^**^p < 0.01, ^***^p < 0.001; Standard errors are in parentheses. Due to space limitation, regression results on control variables were not listed.

**Table 6:** Results of heterogeneity test of the effect of Internet use on depression among older adults.

**Variables**	**Region**		**Gender**	
	**Urban older adults**	**Rural older adults**	**Female older adults**	**Male older adults**
Internet use	−0.088^***^(0 0.024)	−0.151(0 0.035)	−0.093^**^(0 0.032)	−0.064^**^(0.025)
Control variables	Yes	Yes	Yes	Yes
Observations	2,369	2,345	2,240	2,474
R^2^	0.169	0.153	0.154	0.177

^**^p < 0.01, ^***^p < 0.001. Standard errors are in parentheses. Due to space limitation, regression results on control variables are not listed.

**Table 7:** Results of mediating effect based on samples of female and male older adults.

**Types**	**Gender**			
	**Female older adults (*****N*** = **2,240)**		**Male older adults (*****N*** = **2,474)**	
	**Observed coefficient**	**95% Conf. Interval**	**Observed coefficient**	**95% Conf. Interval**
Indirect effect	−0.005 (0.003)	−0.012,0.001	−0.007^**^	−0.0135,−0.001
Direct effect	−0.088^**^(0.029)	−0.146,−0.030	−0.056^*^	−0.105,−0.008
Total effect	−0.093^**^(0.030)	−0.151,−0.035	−0.064^**^	−0.113,−0.015

Adjusting for gender, age, marita status, education attainment, household registration, retirement, party member status, self-assessed socio-economic status, self-assessed social class and physical health. ^*^p < 0.05, ^**^p < 0.01. Confidence intervals do not cross zero means the relationship is significant.

## 4. Discussion

This study confirmed that Internet use could reduce depression among older adults. The result supported some previous findings linking Internet use to lower depression scores (15, 35). The Internet provides social support and information access for individuals, thereby contributing to improvements in older peoples' mental health and social adaptation, such as improving their life satisfaction and happiness, and reducing their levels of depression (36, 37). We also identified the association between individuals' basic demographic characteristics and depression. This study showed that the incidence of depression was related to gender, marital status, education attainment, household registration, retirement, self-assessed socio-economic status, self-assessed social class, and physical health. We found that older men tended to have lower depression scores than older women. And the more educated the older adults, the lower their depression scores. Older adults with spouses had better mental health, and had lower depression scores than those without spouses. Older retirees, who have more free time for leisure activities and no job stress, have a lower risk of depression than non-retired seniors. In addition, higher self-rated socio-economic status, social class and physical fitness were associated with lower depression scores. These results were consistent with some previous results (25, 38). In addition, the relationship between different categories of Internet use and depression among the elderly was explored in this study. Wechat use, short video browsing and online shopping had a protective influence against depression. This conclusion was consistent with part of the findings of Nakagomi et al. (17) and Braun (39), whose findings showed that online communication with family and friends significantly reduced depression among older adults. It also corroborates Yang et al., who reported that online recreational activities were negatively associated with depression (16). While our study obtained a new view that among recreational activities, short video browsing could reduce depression, but online games could not. On the one hand, with the rapid development of short video software, elderly people in China enrich their daily life, relieving loneliness and benefiting their mental health by watching funny and health knowledge videos. On the other hand, the older elderlies may have little interest in online games, and learning online games consume a certain amount of energy and physical strength not conducive to rest and relaxation. Furthermore, online learning had no significant effect on depression among the elderly, which was consistent with the conclusion of some researchers (40). Depression is mostly related to psychological factors. The professional psychological knowledge of the elderly on the Internet was less, and it was difficult for the elderly to relieve their personal emotions and depression through online learning.

Moreover, only older adults in urban areas had statistically significantly lower depression scores when using the Internet. This was because the Internet was developing faster in urban areas than rural areas in China. Urban elderly were more familiar with online communication and online shopping. They were more likely to derive happiness through this means. Further, the reduced effect on depression was more apparent among female older adults than their male counterparts. This was possibly because females are more likely to derive emotional value from online activities such as online communication and shopping, thus increasing their sense of satisfaction and happiness.

Additionally, Internet use affected depression levels among older adults by improving the quality of their relationships. On the one hand, the Internet provides convenient conditions for the elderly to obtain interpersonal relations and maintain and expand their interpersonal network (41). On the other hand, the elderly could also engage in social interaction through the Internet to obtain emotional support and social recognition from others, thus reducing the risk of depression (42). However, the mechanism by which Internet use reduced depression by improving relationships was only found among older men, not women. This was possibly because elderly men are more inclined to use the Internet as a social tool to maintain or expand their social networks and meet their social needs. While elderly women use the Internet to shop and watch short videos to satisfy their consumption and entertainment needs and relieve their negative emotions (43).

## 5. Conclusion

Based on a national sample of Chinese older adults, this study explained the mechanism of how Internet use affects the depression level of older adults. This study unveiled that Internet use could significantly reduce depression levels among elderly individuals, and interpersonal relationship played a mediating role in the relationship between Internet use and depression. Furthermore, only certain types of Internet use (WeChat use, Short Video browsing, Online shopping) were associated with lower depression. Additionally, we found clear demographic differences in the impact of Internet use on depression. In conclusion, this study provides new empirical evidence for the relationship between Internet use and depression among older adults, and enriches the research on the mental health of the elderly in the context of the Internet era.

This study has some limitations. The amount and frequency of Internet use may also be related to depression outcomes, but we did not address this specific issue due to the limitation of data and sample size. We will conduct further analysis in the future.

## Data availability statement

Publicly available datasets were analyzed in this study. This data can be found at: http://www.isss.pku.edu.cn/cfps/.

## Author contributions

YN conceived this research and conducted the first statistical analysis. YX and YH reviewed, edited the manuscript, and were responsible for visualization. All authors discussed paper structure, contributed to different part of the literature, contributed to the article, and approved the submitted version.
